# A 24-well plate assay for simultaneous testing of first and second line drugs against *Mycobacterium tuberculosis* in a high endemic setting

**DOI:** 10.1186/1756-0500-7-512

**Published:** 2014-08-10

**Authors:** Wassihun Wedajo, Thomas Schön, Ahmed Bedru, Teklu Kiros, Elena Hailu, Tesfamariam Mebrahtu, Lawrence Yamuah, Kristian Ängeby, Jim Werngren, Philip Onyebujoh, Kifle Dagne, Abraham Aseffa

**Affiliations:** Armauer Hansen Research Institute, Addis Ababa, Ethiopia; Department of Medical Microbiology, Linkoping University, Linköping, Sweden; Department of Clinical Microbiology MTC, Karolinska Hospital, Karolinska University Hospital, Stockholm, Sweden; Department of Preparedness, Unit of Highly Pathogenic Microorganisms, Swedish Institute for Communicable Disease Control (SMI), Solna, Sweden; Department of Infectious Diseases and Microbiology, Kalmar County Hospital, Kalmar, Sweden; Faculty of Life Sciences, Addis Ababa University, Addis Ababa, Ethiopia; World Health Organization - Regional Office for Africa, Inter-country Support Team for East/Southern Africa, Harare, Zimbabwe; Department of Biology, Jimma University, Jimma, Ethiopia

**Keywords:** Susceptibility testing, Epidemiological cut off value (ECOFF), Multi drug resistant (MDR) tuberculosis, Ethiopia

## Abstract

**Background:**

Early detection of drug resistance is one of the priorities of tuberculosis (TB) control programs as drug resistance is increasing. New molecular assays are only accessible for a minority of the second line drugs and their availability in high endemic settings is also hampered by high cost and logistic challenges. Therefore, we evaluated a previously developed method for drug susceptibility testing (DST) including both first- and second line anti-TB drugs for use in high endemic areas.

**Results:**

Baseline mycobacterial isolates from 78 consecutive pulmonary TB patients from Addis Ababa, Ethiopia who were culture positive for *Mycobacterium tuberculosis* at the end of a two-month directly observed treatment short course (DOTS) were included. The isolates were simultaneously tested for isoniazid, rifampicin, ethambutol, streptomycin, amikacin, kanamycin, capreomycin, ofloxacin, moxifloxacin, ethionamide and para-aminosalicylic acid susceptibility using the indirect proportion method adopted for 24-well agar plates containing Middlebrook 7H10 medium. Applying the 24-well plate assay, 43 (55.1%) isolates were resistant to one or more of the first line drugs tested (isoniazid, rifampicin and ethambutol). MDR-TB was identified in 20.5% of this selected group and there was a perfect correlation for rifampicin resistance with the results from the genotype MTBDR*plus* assay. All isolates were susceptible to aminoglycosides and fluoroquinolones in agreement with the genotype MTBDR*sl* assay. The only tested second line drug associated to resistance was ethionamide (14.1% resistant). The method was reproducible with stable results for internal controls (one multi-drug resistant (MDR) and one pan-susceptible strain (H37Rv) and DST results could be reported at two weeks.

**Conclusions:**

The 24-well plate method for simultaneous DST for first- and second line drugs was found to be reproducible and correlated well to molecular drug susceptibility tests. It is likely to be useful in high-endemic areas for surveillance as well as for the detection of second line drug resistance in targeted groups such as in those who fail empirical MDR treatment.

## Background

Tuberculosis (TB) remains one of the major public health challenges, particularly in high endemic areas where there is a growing and probably underestimated problem of resistance to anti-TB drugs. Early detection of drug resistance is one of the priorities of TB control programs [1]. Management of drug resistant TB has an important impact for improved TB control by employing appropriate multi-drug treatment regimens which are in turn based on the national resistance epidemiology. However, representative and continuous TB drug resistance surveillance data is largely missing in low-income high endemic areas partly due to poor laboratory facilities [2]. A national drug resistance survey in Ethiopia conducted in 2005 reported a multi-drug resistant (MDR)-TB rate of 1.6% among newly diagnosed patients and 11.8% among retreatment cases [3].

The increasing rate of resistance to the commonly used anti-TB drugs causes an urgent need for accurate and reproducible assays for second line drug susceptibility testing (DST) in high endemic areas [4]. Various DST methods have been developed including phenotypic testing based on the growth of the bacilli on drug containing solid or liquid medium [5] and genotypic detection of resistance determining genes [6–8]. Molecular approaches such as line probe assays and the GeneXpert MTB/RIF (Cepheid, Inc. USA) present a significant advantage mainly in terms of rapid diagnosis of MDR-TB and have been recommended as initial diagnostic tests in individuals at risk [7, 9]. A drawback of such molecular based methods is that they include only some of the first and second line anti-TB drugs and that they are not suitable for drug resistance surveillance studies as they only include known drug resistance mutations. In contrast, conventional broth- and agar-based methods have been validated against clinical outcome at least for the first line drugs and are established for most second line drugs used although the breakpoints to predict clinical susceptibility for some of those drugs are not as well characterized [10].

In low income countries, phenotypic DST is commonly performed with conventional methods such as the indirect proportion method on Löwenstein-Jensen (LJ) or Middlebrook 7H10 agar media owing to the relatively low cost. However, most of these conventional phenotypic methods require several weeks to months in order to obtain results. In contrast to phenotypic methods, the genotyping approaches of drug resistance detection are rapid but have limited implementation in daily routine, particularly in resource limited areas where the burden of tuberculosis is substantial, due to their high cost and need for continuous electric power supply [7].

The increasing trend of resistant TB in high endemic areas with insufficient laboratory support [11] is a compelling reason to explore for alternative DST methods for first and second line drugs in patients that are at high risk of developing resistant TB. Effective management of MDR and poly resistant TB under routine program conditions requires assessment of drug resistance to both first and second line drugs to provide the best therapy for the patients and prevent transmission. Automated liquid culture systems, particularly the BACTEC MIGT 960, have been recommended for DST of second line drugs [12]. Implementation of the liquid culture system in resource-constrained settings is, nevertheless, impeded by the high cost of laboratory infrastructures [13, 14].

In this study we applied a previously described low cost DST method which was slightly modified to suit high endemic areas [15]. Our aim was to evaluate this method adapted for 24-well plates containing 7H10 medium for DST of the major first- and second line drugs in Ethiopia.

## Methods

### Patients and strains

A total of 500 newly diagnosed smear positive pulmonary TB patients were enrolled to receive standard therapy against active tuberculosis consisting of isoniazid, rifampicin, pyrazinamide and ethambutol daily under supervision at the treatment center (St Peter’s TB Specialized Hospital, Addis Ababa, Ethiopia). Eighty baseline isolates obtained upon clinical presentation from consecutive patients who were still culture positive at the end of the second month of intensive treatment with the four drugs were used for testing in the 24-well plate assay. From all study participants, written, informed consent was obtained.

### All patients

#### Mycobacterial culture and typing

The Mycobacterial isolates were obtained by sputum culture processed according to standard methods [16] on LJ media (Sigma-Aldrich Chemical Co.) and were confirmed as *Mycobacterium tuberculosis* (Mtb) on DNA isolated from heat killed, culture positive samples using RD9 typing which relies on analysis of species specific genomic deletions [17] and spoligotyping (data not shown). Following genotypic identification, isolates were sub-cultured on LJ and cultures grown within 2-3 weeks were used for susceptibility testing on 7H10 media (Sigma-Aldrich Chemical Co.). Colonies were transferred from LJ to plastic tubes containing 3 ml of distilled water and glass beads (3 mm in diameter). The tubes were shaken for 1 min and the suspension was then allowed to settle for 15 min. The suspension was adjusted to a turbidity equivalent to that of a 1.0 McFarland standard. Subsequently, 24-well plates were inoculated by adding 10 μl of this bacterial suspension.

#### Preparation of drug containing agar series in 24-well plates

In each experimental batch, 10 DST 24-well plates were prepared in the preceding day of inoculation. Thus, a new batch of freshly prepared 24-well plates was used within one day for 13 rounds of DST which included H37Rv and the MDR control isolate. Dilutions of the antibiotics were prepared to get the required antibiotic concentrations. A volume of each antibiotic was mixed with 7H10 medium in 50 ml Falcon tube in a water bath to get the required drug concentration in the agar mixture needed for 10 plates. The drug containing and the drug free media were then manually transferred in 2.5 ml amounts into the respective wells of the 24-well tissue culture plates. The plates were then left in the safety hood until the agar was completely solidified and subsequently sealed with Parafilm and stored at 4°C until used the next day.

#### Drug susceptibility testing with the 24-well plate method

The drug susceptibility patterns of the clinical isolates were determined using a modified indirect proportion method adapted for 24-well agar plates containing 7H10 medium as previously described [15]. Compared to the original method, no automatic dispenser was used and the plates were prepared manually as described. The plates were read at 2 weeks after inoculation by the same evaluator (WW) as it was reported previously that the MIC results could be reported at that time point [15]. Drug susceptibility was read by visual comparison of the drug containing media (1:1 bacterial suspensions) with the drug free control on which 1:100 bacterial suspensions was inoculated. The growth was evaluated according to the proportion method by comparing the 1:100 diluted control to the drug containing wells. The strain was reported susceptible (S) if there was clearly more growth in the 1:100 diluted control than in the drug containing well with the critical concentration and resistant (R) if there was more growth in the drug containing well than in the 1:100 control.

**Figure 1:**
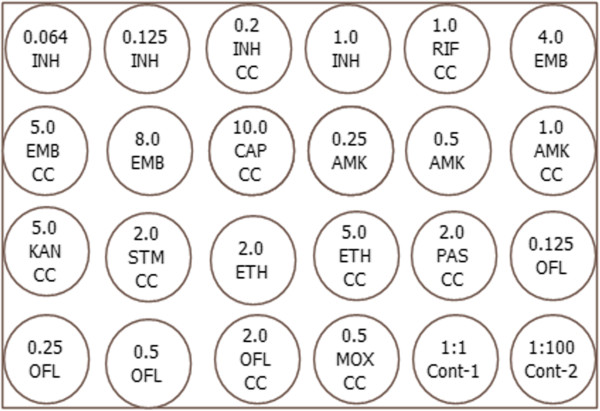
**Outline of the template used for drug concentrations and controls used in the 24-well plate.** The template outlining the distribution and concentrations (μg/ml) of the drugs used is shown. (CC = Critical Concentration, Cont = Control).

The method was also modified for the simultaneous testing of first and second line anti-TB drugs (Sigma-Aldrich Chemical Co. St. Louis, MO, USA) allowing MIC testing for some of the most important drugs (INH, AMK and OFL) currently available in the setting. Additionally, conventional susceptibility testing was based on currently recommended breakpoints (critical concentrations). Thus, the following concentrations were chosen: isonicotinic acid hydrazide (INH), 0.064, 0.125, 0.2 and 1.0 μg/ml; rifampicin (RIF), 1.0 μg/ml; ethambutol (EMB), 4.0, 5.0 and 8.0 μg/ml; streptomycin (STM), 2.0 μg/ml; capreomycin (CAP), 10.0 μg/ml; amikacin (AMK), 0.25, 0.5, 1.0 and 2.0 μg/ml; kanamycin (KAN), 5.0 μg/ml; ethionamide (ETH), 2.0 and 5.0 μg/ml; *p*-aminosalicylic acid (PAS), 2.0 μg/ml; ofloxacin (OFL), 0.125, 0.25, 0.5, 1.0 and 2.0 μg/ml and moxifloaxin (MOX), 0.5 μg/ml [10, 18]. The template used is shown in Figure 1. Drug concentrations other than the critical concentrations were used for some of the above anti-TB drugs for quality control purpose. Growth with the following critical concentrations (CC) of the test drugs defined resistance: 0.2 μg/ml INH, 1.0 μg/ml RIF, 5 μg/ml EMB, 2.0 μg/ml STM, 10.0 μg/ml CAP, 5.0 μg/ml KAN, 5.0 μg/ml ETH, 2.0 μg/ml PAS, 2.0 μg/ml OFL and 0.5 μg/ml MOX [10, 18, 19]. For AMK where no CC was defined by WHO at the time of the study for 7H10 medium, the previously suggested critical concentration based on the wild type distribution was applied (1.0 μg/ml AMK) [19].

#### Hain GenoType MTBDRplus

The evaluation of the 24-well plate method in detecting MDR isolates was also compared with the GenoType MTBDR*plus*. For aminoglycosides and fluoroquinolones the phenotypic results from the 24 well assay was compared to the HAIN MTBDR*sl* assay through the detection of mutations in the *gyrA* and *rrs* genes, respectively. Essentially, the MTBDR*plus* and MTBDR*sl* assays share the same procedure (GenoType® MTBDR*plus*, GenoType® MTBDR*sk* Hain Life science GmbH, Germany). The MTBDR*plus* test is based on PCR amplification of specific regions of RIF and INH resistance conferring genes, *rpoB, katG* and *inhA* followed by reverse hybridization of amplicons to specific probes pre-blotted to membrane strips and colorimetric detection of the key mutations associated with RIF and INH resistance [20].

#### Quality control

Parallel to all 13 sets of test batches, the standard pan susceptible strain, H37Rv (ATCC 27249) where MIC levels have previously been established for the major first- and second line drugs [10, 19, 21] were inoculated on separate plates for all drugs as internal quality control. A clinical MDR-TB strain sent to Ethiopian Health and Nutritional Research Institute (EHNRI) (the national reference laboratory) for proficiency testing with confirmed resistance to INH and RIF was also included as positive control. The critical concentrations applied were according to the recommendations from the WHO [10, 18].

#### Data analysis

Analysis was performed using Graph Pad prism 5. Significant differences in proportions between different groups were assessed using Pearson’s Chi^2^ test with p ≤ 0.05 considered significant.

#### Ethical statement

Approval was obtained from the ethical review committee of the Armauer Hansen Research Institute (AHRI)/All Africa Leprosy, Tuberculosis and Rehabilitation Training Centre (ALERT), Addis Ababa, Ethiopia. Written, informed consent was obtained from all study participants.

## Results

### Quality control

The evaluation of the 24-Well Plate Assay in identifying resistant and susceptible isolates and in determining MIC values of the anti-TB drugs was assured using internal quality controls. The pan susceptible H37Rv (ATCC 27294) strain (n = 13) and one clinical MDR strain (n = 11) were included as internal controls for all drugs and additionally the MIC value of INH, AMK and OFL were evaluated (Table 1). The result showed a very low MIC variation between experiments for H37Rv with one dilution step deviation at two and three experimental occasions for INH and OFL, respectively out of 13 separate experiments and batches of 24-well plates. The same MIC-level at 1.0 μg/ml was detected for AMK in all 13 rounds. Considering the MDR clinical strain used as a control, there was a complete agreement in the MIC level of INH, AMK and OFL and drug susceptibility results in each test round (n = 11) including STM, ETH, CAP, AMK, PAS, KAN and EMB (Table 1). Thus, the reproducibility of the 24-well plate method or H37Rv and the MDR clinical strain for all included drugs was excellent.

**Table 1:** **MIC distributions and intra laboratory variations for isoniazid (INH), amikacin (AMK) and ofloxacin (OFL)**

Test batch	MIC (μg/ml) for H37Rv			MIC (μg/ml) for the QC MDR strain		
	INH	AMK	OFL	INH	AMK	OFL
01	0.125	0.5	0.5	ND	ND	ND
02	0.125	0.5	0.5	ND	ND	ND
03	0.125	0.5	0.5	1.0	1.0	1.0
04	0.125	0.5	0.5	>1.0	1.0	1.0
05	0.125	0.5	1.0	>1.0	1.0	1.0
06	0.125	0.5	1.0	>1.0	1.0	1.0
07	0.125	0.5	1.0	>1.0	1.0	1.0
08	0.125	0.5	0.5	>1.0	1.0	1.0
09	0.125	0.5	0.5	>1.0	1.0	1.0
10	0.2	0.5	0.5	>1.0	1.0	1.0
11	0.2	0.5	0.5	>1.0	1.0	1.0
12	0.125	0.5	0.5	>1.0	1.0	1.0
13	0.125	0.5	0.5	>1.0	1.0	1.0

(ND, no data; QC = Quality control).

### Evaluation of the 24 well DST method for the first line drugs

**Figure 2:**
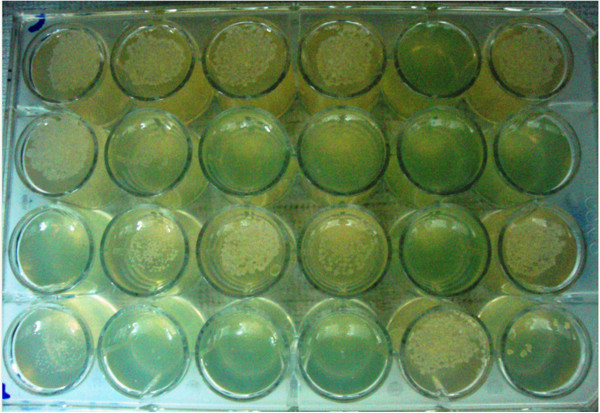
**Example of a typical readout for drug susceptibility testing in the 24-well plate.** Figure 2 shows a typical DST result applying the template used in Figure 1 with a standard inoculum of 10 μl at a turbidity of a 1.0 McFarland standard. This isolate was found to be resistant to INH, EMB, STM and ETH. The MIC for INH is >1.0 μg/ml, for AMK ≤ 0.125 μg/ml and for OFL = 0.25 μg/ml. (CC = Critical Concentration, Cont = Control).

Assessment of drug resistance by the 24-well plate assay was performed on 78 isolates that were confirmed to be Mtb by RD9 and spoligotyping. The remaining two non-tuberculous mycobacterial (NTM) isolates were not included in the final analysis of drug resistance. A representative result from a DST round is presented in Figure 2. The proportion of any type of drug resistance among the clinical isolates was 55.1% (43/78). The highest level of drug resistance (36/78, 46.2%) was observed for STM followed by INH and EMB with a proportion of 39.7% (31/78) and 37.2% (29/78), respectively. MDR-TB was detected in 20.5% of the isolates (16/78). All TB Patient infected with strains resistant to RIF were also resistant against INH and confirmed as MDR-TB.

### Correlation between GenoType MTBDR and the phenotypic DST assay

The evaluation of the 24 well plate method showed full concordance to HAIN MTBDRplus in detecting MDR-TB. However, discrepancies were observed between the two methods in detecting isolated INH drug resistance. Seven more isolates (31/78 *vs* 24/78) were identified as INH resistant by the 24 well plate assay compared to the MTBDR*plus* test (Table 2). Second line drug susceptibility patterns determined by the 24-well plate assay were also compared with GenoType MTBDR*sl* for aminoglycosides and fluoroquinolones. This analysis showed that that all the sixteen MDR isolates were detected as susceptible for these two classes of second line drugs by both tests.

**Table 2:** **Pattern of drug resistance to the major first- and second line drugs**

First line drugs			Second line drugs	
Anti-TB drugs	24-well plate assay	HAIN MTBDR***plus***	Anti-TB drugs	24-well plate assay
INH	31/78 (39.7%)	24/78 (30.7%)	AMK	0
RMP	16/78 (20.5%)	16/78 (20.5%)	KAN	0
EMB	29/78 (37.2%)		CAP	0
STM	36/78 (46.2%)		ETH	11/78 (14.1%)
			PAS	0
			OFL	0
			MOX	0

#### Low prevalence of resistance to 2nd line drugs among patients culture positive at 2 months of treatment

**Figure 3:**
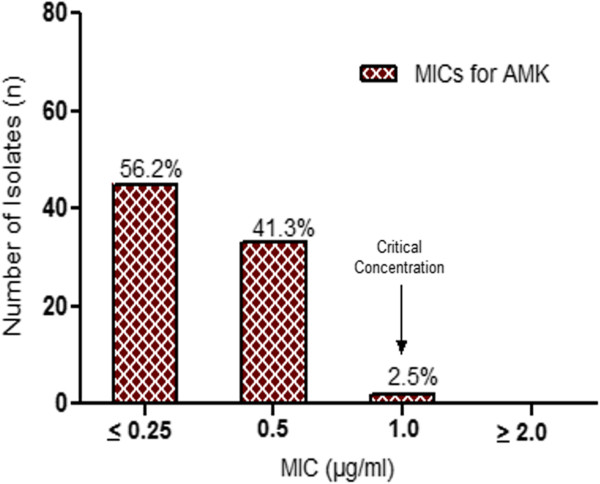
**Minimal inhibitory concentration (MIC) distribution of amikacin.** MIC distributions for the second line drug amikacin among the 78 *Mycobacterium tuberculosis* isolates. (MIC = Minimal inhibitory concentration, AMK = Amikacin.

**Figure 4:**
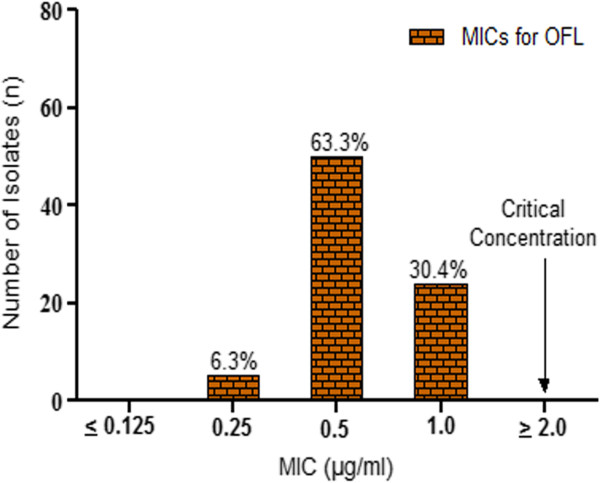
**Minimal inhibitory concentration (MIC) distribution of ofloxacin.** MIC distributions for the second line drug ofloxacin among the 78 *Mycobacterium tuberculosis* isolates. (MIC = Minimal inhibitory concentration, OFL = Ofloxacin.

Resistance to the major second line drugs was also determined together with first line drugs on the 24 well plates. It was found that all isolates including the MDR cases were susceptible to these drugs except for ethionamide where 14.1% of the isolates were resistant. MIC determination of OFL as well as AMK showed that all clinical isolates had MIC levels clearly below the critical concentrations (Figures 3 and 4). Out of the 16 MDR-TB cases, 37.5% were ethionamide resistant. However, no MDR strain was resistant to fluoroquinolones and/or aminoglycosides and thus no XDR-TB was detected which was also confirmed by the MTBDR*sl* assay.

## Discussion

In this study we show the usefulness of the 24-well plate assay for drug susceptibility testing of *Mycobacterium tuberculosis* in resource limited settings. The method is suitable for clinical laboratories with low-cost laboratory materials and reagents and is easily standardized. In our study, test results were available, on average, after 12 days of incubation which is significantly lower than the reporting time in LJ media which is about six weeks [22]. The LJ method is the most widely spread assay for DST in high endemic areas because of its low cost and although the 24-well method is slightly more expensive due to the use of 7H10 medium, it is still less expensive and resource demanding than the routine liquid based and molecular biology based methods. An estimated cost per sample for the 24-well plate assay is 5 Euros per plate where up to 22 drugs could be analyzed [15]. Other previously described low-cost methods suitable for high endemic areas are the microscopic-observation drug susceptibilty (MODS) and thin layer agar (TLA) assays but data for second line drugs are limited [23, 24]. A commonly acknowledged limitation of such assays including the 24-well plate assay is the introduction of increased variation under routine conditions due to lack of the standardization which has been done for routine methods like BACTEC MGIT 960. Rapid molecular techniques are also increasingly available for detecting anti-TB drug resistance. However, such approaches require expensive equipment and reagents which could not be widely applied in high endemic areas and they are limited to commonly known resistance mutations. The 24-well plate method is a phenotypic assay with the potential to determine full DST panels including second line drugs at a substantially lower cost. The assay may also be tailored to suit local treatment traditions in terms of which drugs that are included. In the current study we primarily aimed at including the current critical concentration for the included first- and second line drugs. Secondly we prioritized short MIC-series for important drugs available and in use in the study setting at the time of the study (INH, AMK and OFL) as the MIC level may be important to consider in individualized dosing strategies for patients with XDR TB. Thus, the concentrations used for testing in this study apart from the current critical concentration may need revision according to the availability of the drugs and the national recommendations for the use of second line drugs. In this perspective, MIC testing in future studies should probably be preferred for newer generation fluoroquinolones such as MOX or LEV rather than OFL.

Limitations of our study include the relatively few samples and that only one center was involved. Moreover, the inclusion of quality control strains exhibiting resistance to the second line drugs would be an important step in the development of the method. We find it assuring in terms of quality control and reproducibility for the second line drugs that there was perfect agreement in the H37Rv MIC distribution within 13 separate rounds of testing for the fluoroquinolones and aminoglycosides (Table 1) and a variation within one MIC dilution step compared to previous studies [10, 19, 21]. Both the susceptible control strain (H37Rv) and the MDR clinical strain showed very stable MIC levels for INH, AMK and OFL which indicates a very high stability that is likely also to include slightly higher MIC levels observed in resistant strains. Additionally, the classification of the other drugs was also stable for 13 rounds.

We also evaluated the 24-well plate method with the GenoType MTBDR*plus* in detecting resistance to RIF and INH. The two DST methods had 100% concordance in identifying MDR isolates confirming the overall performance of the 24-well plate method for detection of MDR-TB. However, we observed discrepant DST results between the 24-well plate assay and the Hain MTBDR*plus* test for INH where 7 (9%) of INH resistant isolates at the critical concentration of 0.2 μg/ml were detected as susceptible by the GenoType MTBDR*plus* technique. As previously reported, this is probably due to the known fact that not all genetic alterations causing INH resistance are present in the Hain assay, which also highlights one major drawback of the molecular assays [25]. The GenoType MTBDR*plus* technique could be used as a supplement to the low cost conventional 24-well plate method for MDR case detection [26]. A strategy for combining the use of these techniques could be to employ a rapid molecular assay such as the Hain or GeneXpert in patients who remain smear or culture positive at week 8 in order to rapidly identify MDR cases in need for a rapid shift to an empirical MDR regimen and then use the 24-well assay to screen such isolates for 2nd line drug resistance to provide the best possible definite treatment.

The 24-well method may also be suitable when processing large number of samples like in the case of drug resistance surveillance studies where simultaneous testing of first and second line drugs may be required. It allows producing many plates a day and processing hundreds of isolates. The assay is easily manageable during inoculation, incubation and reading. Overall, it could be an alternative to more resource demanding liquid culture based DST methods. The introduction of the MDR-TB management system in high endemic areas such as Ethiopia makes such assays useful for early identification of multi drug resistance allowing prompt initiation of treatment with subsequent early identification of MDR-TB as a cause of treatment failure.

The proportion of first line anti-TB drug resistance among the study participants is markedly high. Resistance to one or more first line anti-TB drugs was observed in 43 (55.1%) of the 78 consecutive clinical isolates. The overall rate is higher than some of the resistance rates reported from various regions in Ethiopia [27–31]. However, the rate of MDR-TB in our sample of newly diagnosed TB patients (16/500; 3.2%) could be compared to the 2005 national estimate at 1.6% [3]. The discrepancy could be due to the study setting in which our isolates were collected. All the 80 samples were selected from 500 previously untreated cases in total based on culture positivity at two months after taking the first line drug treatment. *In vitro* activities of the second line drugs were also determined simultaneous to the first line drugs. The use of the second line drugs in Ethiopia so far has been low which means that there has been a low selective pressure for second line drug resistance. Our finding revealed that all isolates were susceptible to the major second line drugs except for ETH where we observed 14.1% resistance. A study done in the Northwest region of Ethiopia has also reported the lack of second line drug resistance [30]. In the study of Agonafir [32], ETH resistance was observed in 65.2% of 46 MDR-TB isolates which could be compared to a 37.5% rate of ETH resistance among the MDR-TB cases in the present study. The relatively high rate of ETH drug resistance could partly be due to the occurrence of cross resistance between this drug and INH. In a study done in South Africa, ETH co-resistance was observed in 19 of the 39 INH resistant Mtb isolates [33]. In our report, 41.7% (10/24) of INH resistant cases screened by the 24-well plate assay were also ETH resistant. Over all, the findings on second line drug resistance suggest that so far, there is a very low rate of second line drug resistant isolates in Ethiopia. Inadequately treated patients are at high risk of spreading drug resistant strains. With the introduction of MDR-TB treatment, it is important to continuously screen for second line drug resistance in high endemic areas as the number of cases and the risk of rapidly spreading resistant isolates increases due to inadequate treatment.

## Conclusions

We conclude that the 24 well plate method for simultaneous testing of several first- and second line drugs is a rapid, relatively inexpensive and stable method for the detection of drug resistant *Mycobacterium tuberculosis* isolates.
